# Outcomes of Mastopexy Explantation: A Consecutive Series of 841 Patients

**DOI:** 10.1093/asjof/ojag056

**Published:** 2026-03-27

**Authors:** Elliot M Hirsch

## Abstract

**Background:**

The number of women seeking breast implant removal is increasing. However, there are relatively few studies that evaluate outcomes of breast implant removal and mastopexy.

**Objectives:**

This study evaluates complication and revision rates following simultaneous breast implant removal and mastopexy.

**Methods:**

A retrospective review was performed of 841 consecutive patients who underwent simultaneous implant removal and mastopexy between January 1, 2018 and December 31, 2024 by a single surgeon. Demographic, operative, and prior surgical variables were collected. Complication and revision outcomes were analyzed per patient. Unadjusted associations were assessed using univariate logistic regression to estimate odds ratios (ORs) and 95% CIs. All tests were 2-sided (α = .05).

**Results:**

Complications occurred in 40 patients (4.76%), with a total of 45 complication events. Revisions were performed in 39 patients (4.63%), totaling 40 revision events. In unadjusted analyses, age, BMI, implant size >395 cc, the use of mesh, subglandular plane, history of any mastopexy, history of 2 or more prior augmentations, and history of existing capsular contracture or implant rupture were not statistically significantly associated with complication or revisions. Effect sizes were modest with wide CIs (eg, complications: age per 10 years OR 1.11, 95% CI 0.82-1.50; BMI per 5 kg/m^2^ OR 1.10, 95% CI 0.72-1.67). A history of ≥2 prior mastopexies was associated with significantly increased revision risk (OR 7.72, 95% CI 2.65-22.50). Multivariable modeling was not feasible due to the small number of events relative to covariates.

**Conclusions:**

This study demonstrates that with careful patient selection, simultaneous breast implant removal and mastopexy can be performed safely and effectively. Care should be taken when performing this procedure in patients with a history of 2 or more mastopexy procedures as they may have an elevated risk of revision.

**Level of Evidence: 4 (Therapeutic):**

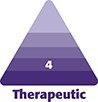

Over the past 30 years, breast augmentation has been one of the most common cosmetic breast procedures, typically averaging over 300,000 cases per year in the United States alone.^1^ It is well known that breast implants have a finite lifespan and typically need to be removed or replaced at some point after implantation.^2^ Therefore, as the population of women with breast implants ages, there is and will continue to be an increasing number of women who require either replacement or removal of their breast implants. Indeed, from 2008 to 2021, the number of women undergoing explantation increased by at least 653%.^3^

Despite an increasing number of women seeking breast implant removal or replacement, there are relatively few studies that evaluate the outcomes of explant procedures, and the existing studies have limitations in terms of small sample size and variability in technique.^4-15^ Therefore, concerns about potential risks and the need to stage procedures persist as well as uncertainties about outcomes. In a previous study, the author described his technique for simultaneous breast implant removal and mastopexy.^16^ The goal of this study is to evaluate the outcomes of this procedure.

## METHODS

This study is a retrospective chart review of a consecutive series of 841 patients who underwent simultaneous mastopexy explantation with a vertical bipedical and open pattern marking technique as described in a previous publication.^16^

### Brief Description of Surgical Technique

Surgical technique begins with induction of anesthesia and injection of tumescent solution into the planned incisions and pedicle. The incisions are made and the pedicle is de-epithelialized. Dissection then proceeds vertically down the lateral and medial borders of the pedicle to the capsule. Capsulectomy is performed as indicated, and the implant is removed. The breast is towel clipped closed, the patient is sat up on the operating room table and adjustments are made. Next, the patient is sat back down, and the areola is tacked superiorly. The pedicle is plicated (either superiorly or inferiorly) and stabilized as needed and then the medial and lateral pillars are closed. Excess inferior skin is trimmed and the incisions are closed.

The guidelines of the Declaration of Helsinki were followed during this study. All procedures were performed by the author between January 1, 2018 and December 31, 2024. Patients who underwent additional simultaneous breast procedures such as fat grafting were excluded from this study. Demographic factors, surgical factors, specific information about prior procedures, as well as complications and revisions were recorded.

Patients with any level of nicotine intake were instructed to stop smoking 3 months prior to performing this procedure. Patients with a significant amount of breast tissue, often with BMI >30 kg/m^2^, were required to lose weight prior to surgery. All patients underwent capsulectomy procedures, the goal of which was a total intact capsulectomy meaning removal of the implant and capsule as a single unit.^17^ When this was not technically feasible, patients underwent a capsulectomy with excision of as much of the capsule as was safely possible. When the posterior capsule could not be safely removed, it was cauterized. Indications for capsulectomy were patient preference, with the exception of capsular contracture or ruptured implant cases. Drains were used in all cases.

### Statistical Analysis

Descriptive statistics summarized patient demographics, prior surgical history, and operative variables. Univariate logistic regression was used to estimate odds ratios (ORs) and 95% CIs for complications and revisions. Continuous predictors were scaled (age per 10 years, BMI per 5 kg/m^2^). In addition, implant size cutoff was >395 cc (approximately 400 cc), a clinically relevant threshold allowing balanced group sizes. Categorical variables were chosen for analysis based on theoretical effect on complication/revision rates. Analysis were 2-sided, with α = .05. Complication and revision rates were calculated per patient (presence of ≥1 event). Total events were also reported. Missing data were handled by variable-wise complete-case analysis; missingness for implant size is detailed where appropriate. A multivariable model was not performed due to the limited number of events (40 patients with complications, 39 with revisions), which would result in overfitting.

## RESULTS

A total of 841 patients (1682 breasts) were included in this study. Demographic information and information regarding prior breast procedures is shown below in Table 1. Information regarding prior breast procedures is shown below in Table 2.

**Table 1.** Information About Prior Breast Procedures

Demographic information and information about prior procedures	
Average age in years (range, SD)	48 (24-84, 10.26)
Average BMI kg/m^2^ (range)	24.5 (15.9-39.1, 3.70)
Mean time from last procedure to explant in years (range, SD)	15.4 (1-50, 8.2)
Average follow-up time in months (range, interquartile range)	5.5 (1 week-64 months, 5 months)
Prior breast operations	*n* (%)
Prior incisions	
Periareolar incision	273 (32.5%)
Wise pattern incision	221 (26.3)
Inframammary incision	214 (25.4%)
Circumareolar incision	63 (7.5%)
Transaxillary incision	41 (4.8%)
Inframammary and periareolar or circumareolar incision	23 (2.7%)
Other	6 (<0.007%)
Number of patients with 1 prior augmentation	610 (72.5%)
Number of patients with 2 prior augmentations	156 (18.5%)
Number of patients with 3 prior augmentations	45 (5.4%)
Number of patients with 4 or more prior augmentations	30 (3.6%)
Number of patients with 1 prior wise pattern mastopexy	201 (23.9%)
Number of patients with 2 or more prior Wise pattern mastopexy	20 (2.4%)
Prior silicone implants	412 (49%)
Prior saline implants	429 (51%)
Average implant size (range)	395 cc (100-900 cc)
Prior subglandular implant placement	134 (16%)
Prior submuscular/dual plane implant placement	707 (84%)

**Table 2.** Indications for Explantation

Indications for explantation	*n* (%)
SSBI or health concerns related to implants	435 (51.7%)
No longer wants implants	166 (19.7%)
Ruptured implants	70 (8.3%)
Grade III/IV capsular contracture	62 (7.3%)
Breast pain	38 (4.5%)
Implants too large	35 (4.2%)
Not specified	14 (1.7%)
Cosmetic concerns other than size	7 (<1%)
Recalled implants	5 (<1%)
Seroma	4 (<1%)
Oncoplastic reconstruction for breast cancer	3 (<1%)
Symmetry after lumpectomy (one side radiated)	2 (<1%)

SSBI, systemic symptoms with breast implants or other health concerns related to breast implants.

A total of 40 patients (4.76%) experienced at least one complication, accounting for 45 complication events (4 patients experienced more than one event). Revisions were performed in 39 patients (4.63%), for a total of 40 revision events (one patient experienced 2 events). The distribution of specific complications and revision types appears in Table 3.

**Table 3.** Complications and Revisions

Complications	*n* (%)
Delayed healing >30 days requiring operative closure	0 (0%)
Delayed healing >30 days, resolved with dressings^a^	12 (1.43%)
Hematoma	6 (0.71%)
Infection requiring intravenous antibiotics	1 (0.11%)
Infection requiring oral antibiotics	15 (1.78%)
Partial or total nipple/areola necrosis	0 (0%)
Retained surgical drain	1 (0.11%)
Seroma requiring drain placement	10 (1.19%)
Revisions	
Cosmetic revision^b^	10 (1.19%)
Dog ears requiring revision^c^	13 (1.55%)
Excision of residual areola tissue on vertical limb	8 (0.95%)
Hypertrophic scarring requiring revision	9 (1.07%)

^a^All were delayed healing at the T junction, except for one patient who experienced skin necrosis on the medial breast flap adjacent to the T junction.

^b^Two cosmetic revisions were performed in the operating room, 8 were performed in the office under local anesthesia and consisted of improving symmetry, minor inframammary revision, correcting small contour defects, minor fat grafting procedures, and minor areola revision.

^c^Eleven dog ear revisions were in the first 250 patients. After adjustment of technique, 2 additional patients required dog ear revision.

Univariate logistic regression analyses evaluating associations between patient and operative factors with complications and revisions are summarized in Table 4. When analyzed as continuous variables, neither age (per 10-year increment) nor BMI (per 5 kg/m^2^ increment) was significantly associated with complications or revisions. For complications, the OR for age was 1.11 (95% CI, 0.82-1.50) and for BMI was 1.10 (95% CI, 0.72-1.67). For revisions, the OR for age was 0.81 (95% CI, 0.59-1.13) and for BMI was 0.65 (95% CI, 0.40-1.06). None of the categorical variables including implant size >395 cc, use of mesh, subglandular implant positioning, history of capsular contracture or implant rupture, any prior mastopexy, or a history of 2 or more prior augmentation procedures demonstrated statistically significant associations with either complications or revisions.

**Table 4.** Odds Ratio Calculations for Variables of Interest

	No complications (*N* = 801)		Complications (*N* = 40)		Complication risk			No revision (*N* = 802)		Revision (*N* = 39)		Revision risk		
	*N*	%	*N*	%	OR	95% CI	*P*	*N*	%	*N*	%	OR	95% CI	*P*
Age per 10 years					1.11	0.82-1.50	.52					0.81	0.59-1.13	.22
BMI per 5 units					1.10	0.72-1.67	.66					0.65	0.40-1.06	.08
Implant size >395 cc^a^	246	47.8%	10	52.6%	1.22	0.49-3.04	.68	237	47.1%	19	61.3%	1.78	0.85-3.74	.13
History of 2+ augmentations	217	27.1%	15	37.5%	1.62	0.84-3.12	.15	218	27.2%	14	35.9%	1.50	0.77-2.94	.24
Use of mesh	18	2.2%	2	5.0%	2.29	0.51-10.2	.28	18	2.2%	2	5.1%	2.35	0.53-10.5	.26
Subglandular implant placement	131	16.4%	3	7.5%	0.42	0.13-1.37	.15	130	16.2%	4	10.3%	0.59	0.21-1.69	.33
History of capsular contracture	59	7.4%	3	7.5%	1.02	0.31-3.41	.98	59	7.4%	3	7.7%	1.05	0.31-3.51	.94
History of implant rupture	66	8.2%	4	10.0%	1.24	0.43-3.58	.70	67	8.4%	3	7.7%	0.91	0.27-3.05	.88
History of Mastopexy	214	26.7%	7	17.5%	0.58	0.25-1.34	.20	210	26.2%	11	28.2%	1.11	0.54-2.26	.78
History of 2 or more mastopexy	18	2.2%	2	5.0%	2.29	0.51-10.2	.28	15	1.9%	5	12.8%	7.72	2.65-22.5	.00018

^a^Missing for 226 among those with no complications, 19 for those with complications, 238 for those with no revision, and 7 for those with revision.

The only variable significantly associated with an increased risk of revision was a history of 2 or more prior mastopexies. Patients in this group demonstrated an OR of 7.72 (95% CI, 2.65-22.5) for undergoing a revision compared with patients who had undergone one or fewer mastopexy procedures. Although this subgroup also showed a higher OR for complications (OR, 2.29; 95% CI, 0.51-10.2), this association was not statistically significant. Odds ratio calculations for the variables of interest are shown in Table 4.

**Figure 1.**
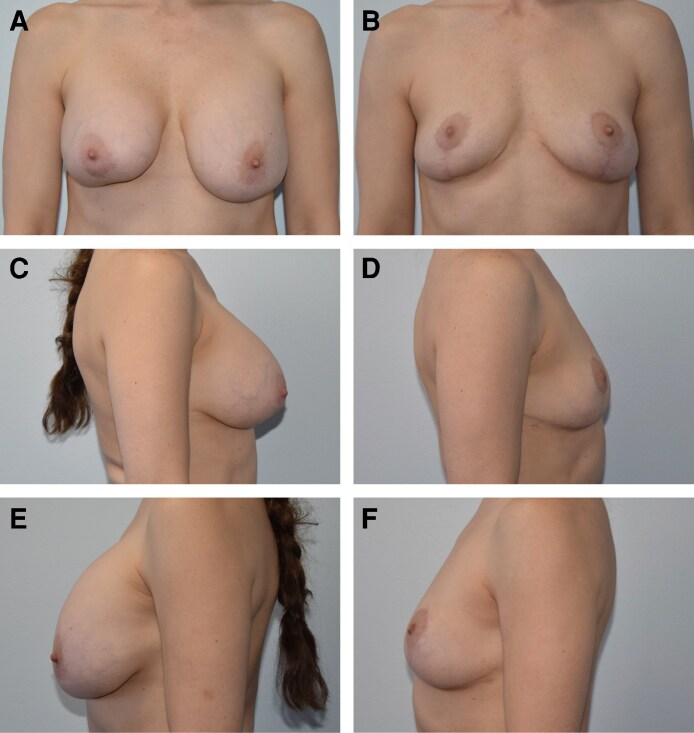
Forty-four-year-old female, with 18-year history of bilateral 350 cc silicone breast augmentation. She developed right grade III capsular contracture. A) Preoperative AP view. B) Same patient, 10 months after undergoing bilateral simultaneous breast implant removal, total complete capsulectomy, and mastopexy with vertical bipedicle and open pattern marking technique, postoperative AP view. C) Preoperative right lateral view. D) Postoperative right lateral view. E) Preoperative left lateral view. F) Postoperative left lateral view.

**Figure 2.**
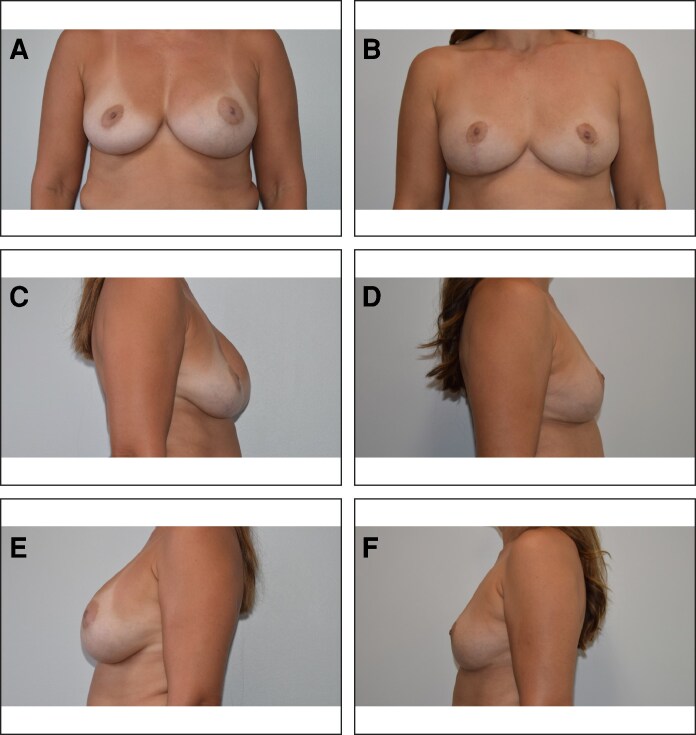
Forty-two-year-old female, with 18-year history of bilateral 425 cc saline breast augmentation. The right implant ruptured 4 months prior to surgery. A) Preoperative AP view. B) Same patient, 14 months after undergoing bilateral simultaneous breast implant removal, total complete capsulectomy, and mastopexy with vertical bipedicle and open pattern marking technique, postoperative AP view. C) Preoperative right lateral view. D) Postoperative right lateral view. E) Preoperative left lateral view. F) Postoperative left lateral view.

**Figure 3.**
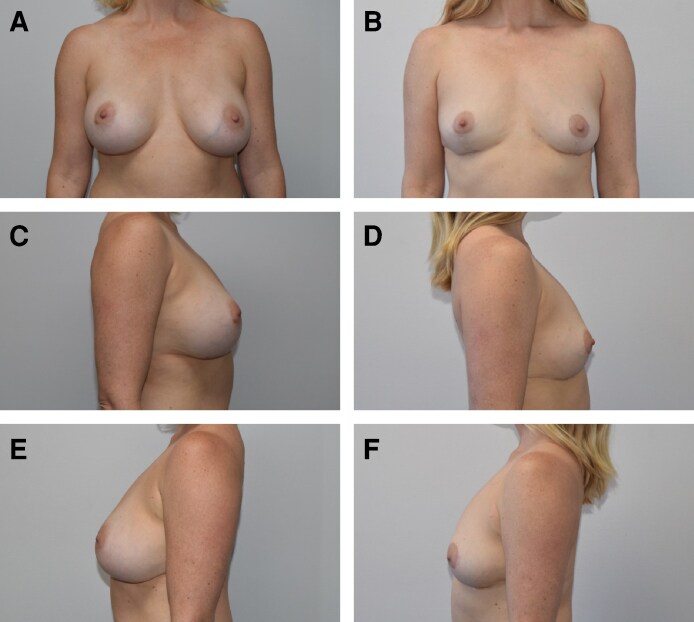
Forty-one-year-old female, with 5-year history of bilateral 375/400 cc silicone breast augmentation. A) Preoperative AP view. B) Same patient, 15 months after undergoing bilateral simultaneous breast implant removal, total complete capsulectomy, and mastopexy with vertical bipedicle and open pattern marking technique, postoperative AP view. C) Preoperative right lateral view. D) Postoperative right lateral view. E) Preoperative left lateral view. F) Postoperative left lateral view.

In addition, 8 patients (<1%) elected to undergo secondary augmentation with implants, and twenty patients (2.6%) elected to undergo secondary fat grafting to increase overall volume (Figures 1-3).

## DISCUSSION

In a previous publication, the author described his technique for performing simultaneous breast implant removal and mastopexy. This study is currently the largest published study of complication and revision rates following breast implant removal and mastopexy. Using patient-level definitions, the overall complication rate was 4.76% and overall revision rate was 4.63%. This study shows that simultaneous breast implant removal and mastopexy can be performed safely and effectively.

Of special note, this study demonstrates a zero incidence of partial or total nipple–areola complex necrosis, which is a significant potential risk in this type of procedure. Multiple prior incisions, unknown pedicles, and the unknown dissections performed previously can all potentially reduce blood flow to the nipple–areolar complex. One theoretical benefit of a vertical bipedicle is that it incorporates both a superior pedicle as well as an inferior pedicle and also provides an alternate pathway for blood flow in the event of a prior inframammary or periareolar incision. Given the zero incidence of partial or total nipple–areola complex necrosis, this study demonstrates that a vertical bipedical and open pattern marking technique safely preserves blood flow to the nipple–areolar complex.

Another advantage of a vertical bipedicle is that it gives excellent exposure to perform a capsulectomy. In the technique evaluated in this study, the explant/capsulectomy was performed through vertical incisions along the medial and lateral borders of the pedicle, not through an inframammary approach. Because of the amount of exposure, it is possible to perform any desired type of capsulectomy without altering external incision size given that the length of external incisions is determined by the amount of skin excess not the capsulectomy type. When considering the effect of capsulectomy type on blood flow to the nipple–areola complex, it is important to note that a total capsulectomy has the identical endpoint as a total intact capsulectomy; the implant is removed and all (or as much as possible) of the capsule is removed as well. The extent of dissection is therefore identical because of the exposure afforded by the vertical approach, so it would not be expected that capsulectomy type would affect complication or revision rates in the context of simultaneous explant/mastopexy using the technique described in this paper.

Age and BMI were analyzed as continuous variables and neither demonstrated a statistically significant association with complications or revisions. However, CIs were wide due to the low number of events, and these findings should not be interpreted as evidence of no association. Importantly, higher- BMI patients in this series were carefully selected, patients with elevated BMI and substantial native breast tissue were required to lose weight preoperatively to enable adequate vertical pedicle mobility and reduce dead space. As a result, the subset of higher-BMI patients included here had relatively lower tissue volume than typically seen at these BMI levels. Therefore, the absence of a BMI signal reflects selective eligibility rather than generalizable safety across all higher-BMI patients.

When considering indications for revision procedures, dog ear excision was found to be the most common (13 patients, 1.5%). Of note, 11 of these dog ear revisions were in the first 250 patients (4.4%), and only 2 additional dog ear revisions were performed in the next 561 patients (<1%). The decrease in dog ear revisions was due to a technical adjustment that the author made. Initially, the lateral extent of the incision was kept as short as possible in order to avoid a visible lateral scar. However, it was noted that small lateral dog ears do not “settle,” and tend to persist after surgery. The author then adjusted the surgical technique by lengthening the lateral incision in order to remove lateral dog ears intraoperatively, and the dog ear revision rate decreased.

Although difficult to quantify objectively, the quality of patient skin was a critical factor in achieving a successful aesthetic result. In the procedure described in this study, poor skin quality or tissue laxity caused the aesthetic result to be less long lasting, leading to pseudoptosis or distortion in areola shape as the skin settled. While there was no objective preoperative measure of skin quality or laxity used in this study, it was noted that patients with 2 or more prior Wise pattern mastopexy procedures had a statistically significant increase in revision rates (OR 7.72, 95% CI 2.65-22.5). The most likely explanation for this is that these patients likely had poor skin quality or laxity, which led them to undergo multiple mastopexy procedures prior to their explant. Because removing a breast implant inherently involves the creation of skin excess, it could be inferred that larger implants would create a larger amount of skin excess and therefore be more likely to require revision. Interestingly, implant size >395 cc did not result in increased complication or revision rates, which implies that larger amounts of skin excess can be effectively managed with the technique described in this study, whereas the intrinsic quality of the skin is the more important determinant of need for revision.

This study has several limitations. Its retrospective design introduces potential selection and information biases, and all analyses are unadjusted. The procedures were performed by a single surgeon, so results may not be generalizable. Only 40 patients experienced complications and 39 experienced revisions, precluding multivariable modeling due to risk of overfitting; consequently, residual confounding remains possible, and wide CIs reflect imprecision due to the low event rate. Missing data for implant size required complete-case analysis and may have introduced bias if data were not missing at random. The average follow-up of 5.5 months may underestimate late events. Additionally, objective aesthetic outcomes such as validated patient-reported measures or blinded photographic assessments were not collected and will be addressed in future prospective studies.

## CONCLUSIONS

This study is currently the largest published series that evaluates the outcomes of simultaneous mastopexy and explantation. With an overall complication rate of 4.76%% and overall revision rate of 4.63%, this study shows that simultaneous breast implant removal and mastopexy can be performed safely and effectively. Careful consideration should be given to performing this procedure in patients with a history of 2 or more mastopexies. Future prospective studies will objectively evaluate cosmetic outcomes.
